# Decrease of 5-hydroxymethylcytosine in primary cutaneous CD4^+^ small/medium sized pleomorphic T-cell lymphoproliferative disorder^⋆^

**DOI:** 10.1016/j.abd.2023.01.003

**Published:** 2023-08-30

**Authors:** Jiahui Hu, Xinyue Zhang, Lihong Zhao, Qiang Zhao, Songmei Geng

**Affiliations:** Department of Dermatology, The Second Affiliated Hospital, School of Medicine, Xian Jiaotong University, Xian, China

**Keywords:** Cutaneous T-cell lymphoma, DNA methylation, Epigenomics

## Abstract

**Background:**

Primary cutaneous CD4^+^ small/medium-sized pleomorphic T-Cell lymphoproliferative disorder (PC-SMTLD) has been considered as a controversial dermatological disease that has been included in cutaneous T-cell lymphoma group, presenting most commonly as a solitary nodule and/or plaque with a specific and characteristic head and neck predilection. Due to the considerable overlap between PC-SMTLD and pseudolymphoma (PL), the differential diagnosis is often challenging. Methylation of DNA at position 5 of cytosine, and the subsequent reduction in intracellular 5-hydroxymethylcytosine (5-hmC) levels, is a key epigenetic event in several cancers, including systemic lymphomas. However, it has rarely been studied in cutaneous lymphomas.

**Objectives:**

The authors aimed to explore the role of differential 5-hmC immunostaining as a useful marker to distinguish PC-SMTLD from PL.

**Methods:**

Retrospective case series study with immunohistochemical and immunofluorescence analysis of 5-hmC was performed in PL and PC-SMTLD.

**Results:**

Significant decrease of 5-hmC nuclear staining was observed in PC-SMTLD when compared with PL (p < 0.0001). By semi-quantitative grade integration, there were statistical differences in the final 5-hmC scores in the two study groups. The IF co-staining of 5-hmC with CD4 revealed a decrease of 5-hmC in CD4^+^ lymphocytes of PC-SMTLD.

**Study limitations:**

The small clinical sample size of the study.

**Conclusions:**

The immunorreactivity of 5-hmC in CD4^+^ lymphocytes was highly suggestive of a benign process as PL. Furthermore, the decrease of 5-hmC nuclear staining in PC-SMTLD indicated its lymphoproliferative status and helped to make the differential diagnosis with PL.

## Introduction

Primary cutaneous CD4^+^ Small/Medium Sized Pleomorphic T-Cell Lymphoproliferative Disorder (PC-SMTLD) has emerged as a distinct clinicopathological entity, presenting most commonly as a solitary nodule and/or plaque with a characteristic head and neck predilection.^1^ Histologically, PC-SMTLD was characterized by a nodular to diffuse infiltration of small to medium-sized pleomorphic T cells (CD3^+^, CD4^+^, CD8^−^, and CD30^−^) throughout the entire dermis, with a small number (<30%) of large CD4^+^ pleomorphic T-cells, the epidermis is uninvolved. In most cases, there were fairly large mixture with small reactive CD8^+^ T-cells and CD20^+^ B-cells, including some histiocytes, neutrophils, plasma cells, and eosinophils.^2^ PC-SMTLD used to be called primary cutaneous CD4^+^ T-cell lymphoma at the WHO (2008) European Organization for Research and Treatment of Cancer. However, because of its inert biological behavior and uncertain malignant potential, the entity was redesignated as “primary cutaneous CD4-positive small/medium sized pleomorphic T-cell lymphoproliferative disorder” in the 2016 WHO classification scheme.^3^

By far, the most important and difficult diagnostic consideration involves benign lymphoid hyperplasia.^4^ An important entity in the realm of cutaneous lymphoid hyperplasia is Pseudolymphoma (PL). PL is characterized by red or brown papules, plaques or subcutaneous nodules of the head, neck, and upper limbs. The pathological features were nodular infiltration or diffuse infiltration of lymphocytes in the middle and lower dermis, mainly small lymphocytes, accompanied by histiocytes, eosinophils, and plasma cells. There may be lymphoid follicles and germinal centers. PL ultimately behaves in a clinically benign fashion, the lesions eventually resolve, either spontaneously, after removal of an identified, initiating agent or with nonaggressive therapy (such as topical use or intradermal injection of steroids).^5^

In many cases, it may be impossible to distinguish between them.^6^, ^7^ PC-SMTLD generally has a favorable prognosis and indolent behavior especially with solitary lesions, the 5-year survival rate is 98.4% among reported cases. While few patients whose outcomes are probably unfavorable may present with an aggressive course, multiple, large and rapidly growing, recurrent, treatment-resistant, and fatal lesions.^8^ In this instance, it may be better to distinguish PC-SMTLD from PL so that the authors can continue vigilant clinical follow-up or choose different treatments.

5-hydroxymethylcytosine (5-hmC) is the oxidation product of 5 methylcytosine (5-mc), which is also an important epigenetic marker involved in various physiological and pathological processes. 5-hmC is catalyzed by the 5-mC hydroxylases: Ten-Eleven Translocation Enzyme (TET) family, including TET-1, -2 and -3. The Ten-Eleven Translocation Enzyme-2 (TET2) has been extensively studied in epigenetic events. The Gene Ontology database showed that most of the genes changed by abnormal methylation were related to gene transcription regulation and cell metabolism. As an important intermediate product of DNA demethylation, 5-hmC has recently been found only in mammals, with deletion expression in various cancers, suggesting that a decrease of 5-hmC is one of the important epigenetic events occurring during cell differentiation and tumor development.^9^, ^10^, ^11^ The expression of 5-hmC has been shown to be a potential marker for differentiating malignant melanoma from benign nevi.^12^

The authors hypothesized that 5-hmC could be a potentially useful biomarker to distinguish PC-SMTLD from CD4^+^ benign cutaneous lymphoid hyperplasia (PL). Moreover, the authors aim to depict the profile of the 5-hmC in cutaneous T-cell lymphoproliferative diseases.

## Materials and methods

### Patient cohorts and tissue samples

A total of 6 cases of PC-SMTLD and 20 cases of PL diagnosed in dermatology department from January 2012 to June 2022 were enrolled for immunohistochemical analysis, all the patient data were anonymized. Informed consent was obtained from each patient before the surgery and all specimens were clearly diagnosed by at least two senior dermatologists. The inclusion criteria for the PC-SMTLD and PL groups were clinically and pathologically based on the criteria defined by the World Health Organization-European Organization for Research and Treatment of Cancer (WHO-EORTC) classification.^13^, ^14^, ^15^

### Immunohistochemistry (IHC) staining

Immunohistochemical studies were performed according to regular procedure (antibody to 5-hmC, diluted in 1:5000, Active Motif; antibodies to TET-2, diluted in 1:500, Abcam). In addition, appropriate positive controls were included. Normal tonsil tissue from otolaryngology was selected as the external positive control. Expression of 5-hmC and TET2 in macrophages and dendritic cells in tonsils were used as positive controls. Negative controls were obtained by omitting the primary antibody.

### Immunofluorescence staining

Immunofluorescence analysis was performed according to regular procedure (antibodies to 5-hmC, diluted in 1:500, Active Motif; antibodies to CD4, diluted in 1:200, Santa Cruz). Appropriate isotype-matched antibody was used for all experiments. Imaging was performed using a C_2_ confocal microscope connected to a digital camera (Nikon, Japan).

### Scoring system

After immunohistochemical staining, two pathologists reviewed all the tissue sections and confirmed the staining results, the pathologists were blinded when assessing 5-hmC. 5-hmC positive staining showed brown-yellow granules, mainly located in the nucleus. The 5-hmC positive cell rate and color intensity of each section were graded by a semi-quantitative integral method.^16^ Dyeing intensity was divided into 4 grades: 0 for no staining, 1 for light coloring, 2 for medium yellow, and 3 for brown. In addition, the percentage of 5-hmC positive cells was assessed using representative High-Power Fields (HPF) (/μm^2^). A total of 5 non-overlapping HPF were randomly selected to count the percentage of 5-hmC positive cells in each histologic section. The final 5-hmC score of each slice was the product of the dyeing intensity score and percentage of 5-hmC positive cells: ≥4 points is high expression, < 4 points is low expression.

### Statistical analysis

Statistical analyses were performed using GraphPad Prism 9.0. Images were processed by Image J (NIH, USA). Unless specifically mentioned, a two-tailed Student’s *t*-test was used to compare differences between the two groups; p < 0.05 was taken as the threshold for significant differences. All data were shown as mean ± S.D.

## Results

### Clinical features of PC-SMTLD and PL

The study groups were comprised of 14 male and 12 female patients with a median age of 48.5 years at presentation (range, 17 to 83y). No patients were deceased at the time of data collection. Extra cutaneous diseases were excluded by staging according to international guidelines.^15^ The diagnosis of PC-SMTLD and PL was based on clinicopathological correlation. All cases showed the characteristic histological features, as previously described. Routine laboratory tests and images (superficial lymph nodes and chest X-Ray) were evaluated in every case, all the investigations were negative, and no patients had a history of clinical lesions suggestive of mycosis fungoides. In addition, no relevant comorbidities emerged during the follow-up period (Table S1, Table S2).

### Histological and immunohistochemical findings of PC-SMTLD and PL

**Figure 1:**
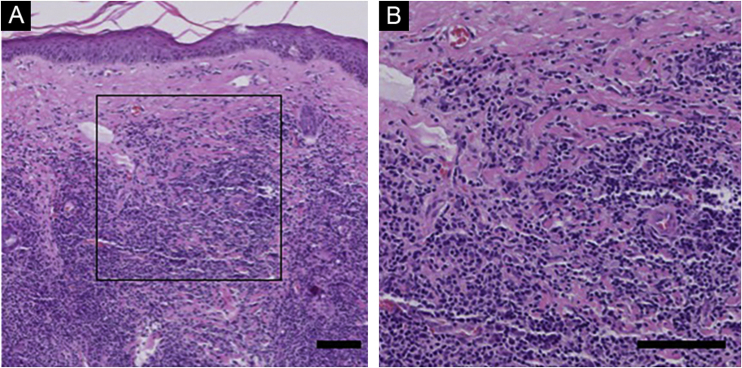
**Histological Findings of PC-SMTLD**. No obvious abnormalities were observed in the epidermis and diffuse lymphoid cell infiltration was observed in the entire dermis. At high magnification, a large number of small to medium sized lymphocytes were seen in the dermis, with mast cells, plasma cells, and eosinophils scattered in the background. (Hematoxylin & eosin, scale bar = 100 μm).

In PC-SMTLD, all cases showed a nodular-to-diffuse infiltrate throughout the entire dermis, even extending into the subcutaneous adipose tissue, the epidermis was uninvolved (Fig. 1). The infiltrates were predominantly composed of small/medium-sized lymphocytes with variable numbers (5%–20%) of medium-sized to large lymphoid cells with hyperchromatic nuclei. In all cases, there was a considerable admixture with histiocytes, which in some cases were accompanied by multinucleated giant cells. Plasma cells were generally present, but eosinophils were few.

In PL, most cases showed predominantly intradermal, dense, lymphocytic infiltrates (nodular or diffuse). It extended to the subcutis in some cases. In general, the infiltrate consists of small to medium-sized B-lymphocytes without significant nuclear atypia. Reactive germinal centers or lymphoid follicles (Fig. S1) are commonly found.

**Figure 2:**
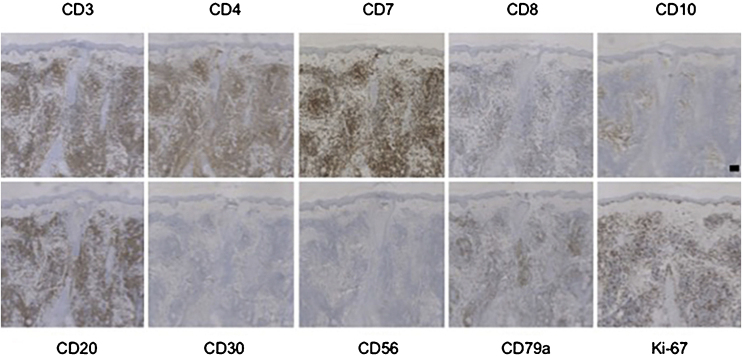
**Immunohistochemistry of PC-SMTLD**. Case 2. The neoplastic cells strongly express CD3, CD4 and CD7; rarely express CD8, CD10 or CD30, do not express CD56. (IHC, scale bar = 100 μm).

Immunohistochemistry showed in all PC-SMTLD cases positivity for CD3 and CD4. CD7 was positive in all tested cases. CD8^+^ lymphocytes were few or absent. CD30 were all negative in all 6 cases and no cases were CD56^+^. The positive TIA-1 was few, only one case showed expression of TIA-1. N/A, Not available (Fig. 2, Table S3).

### Immunohistochemical analysis

**Figure 3:**
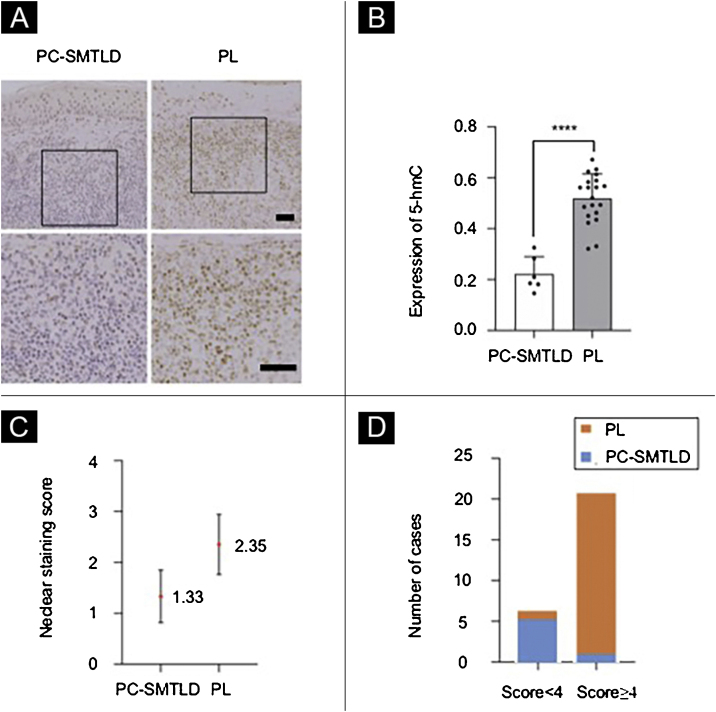
**Expression of 5-hmC**. primary cutaneous CD4^+^ small/medium sized pleomorphic T-cell lymphoproliferative disorder (PC-SMTLD) and Pseudolymphoma(PL): significant decrease of 5-hmC expression was observed in PC-SMTLD when compared with PL in dermis (Fig. 3A). (****p < 0.0001). (Fig. 3B). The difference in nuclear staining score for 5-hmC was statistically significant with higher scores in the PL group (mean = 2.35, Standard Deviation [SD = 0.50]) when compared to PC-SMTLD (mean = 1.33, SD = 0.47, p = 0.0014) (Fig. 3C). The majority of PL 95% (19/20) cases were in the high expression group (Fig. 3D).

All 26 cases demonstrated a distinct strong nuclear staining for 5-hmC in the epidermis (Fig. 3A), while the staining in infiltrated lymphocytes was diverse. The mean positive expression rate of 5-hmC was 22.14% (95% CI 22.14%±4.99%) in 6 cases of PC-SMTLD and 51.43% (95% CI 51.88%±4.10%) in 15 cases of PL. A significant decrease of 5-hmC expression was observed in PC-SMTLD when compared with PL (p < 0.0001) (Fig. 3B).

**Figure 4:**
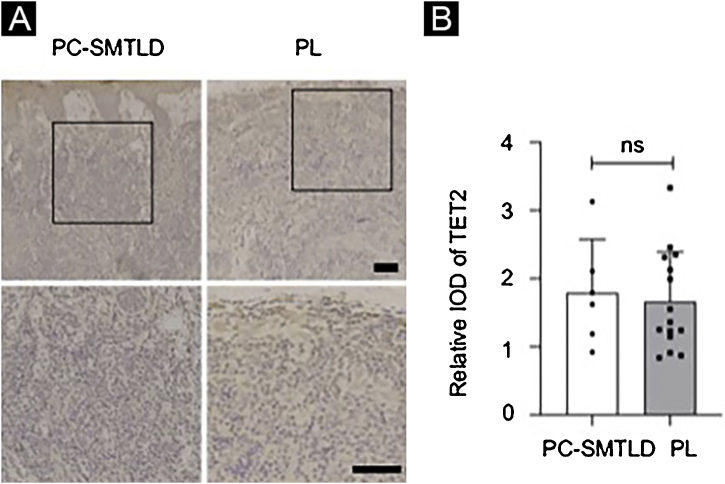
**Expression of TET2**: No significant difference in TET2 expression were observed in two groups.

According to the dyeing intensity score standards and semi-quantitative integral method mentioned above, a 5-hmC nuclear staining score can be obtained. The difference in nuclear staining score for 5-hmC was statistically significant with higher scores in the PL group (mean = 2.35, Standard Deviation (SD = 0.50) when compared to PC-SMTLD (mean = 1.33, SD = 0.47, p = 0.0014) (Fig. 3C). The majority of PL 95% (19/20) cases were in the high expression group (Fig. 3D). No significant difference in TET2 expression were observed in two groups (Fig. 4).

### Immunofluorescence analysis

**Figure 5:**
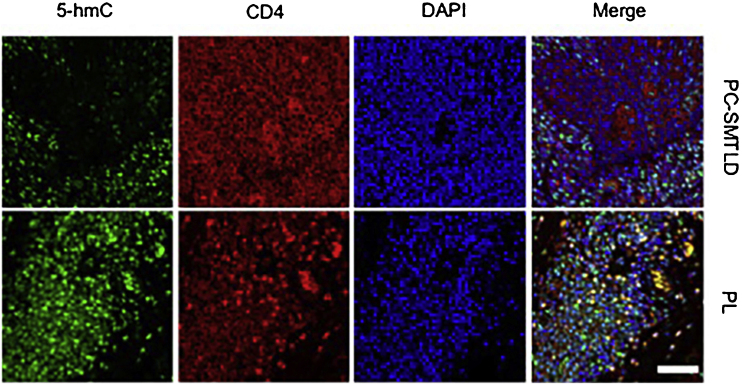
**Immunofluorescence (IF) co-staining of 5-hmC (green) and CD4 (red) in PC-SMTLD and PL samples**: Decrease of 5-hmC in CD4^+^ lymphocytes were observed in PC-SMTLD. DAPI (blue) was used to show cell nucleus.

Considering that the infiltrating lymphocytes in lesions consist of both neoplastic T-cells and reactive lymphocytes, the authors performed Immunofluorescence (IF) double staining of 5-hmC and CD4 to examine the cell source of 5-hmC reduction. As expected, the 5-hmC reduction occurred in CD4^+^ T-cells (Fig. 5).

## Discussion

The authors identified alterations in the epigenetic pattern related to DNA methylation in PC-SMTLD. Specifically, the authors found that 5-hmC expression was highly preserved in PL, while there was a significant decrease of 5-hmC expression in PC-SMTLD. In systemic lymphomas, the downregulation of 5-hmC expression appears to be a key event for tumorigenesis.^9^, ^17^ The authors speculate that this may also be the case for cutaneous CD4^+^ small/medium-sized pleomorphic T-cell lymphoproliferative disorders. Alternatively, the decreased expression of 5-hmC could represent an epiphenomenon secondary to a distinct pathway leading to carcinogenesis.^18^, ^19^ Therefore, further studies will be critical for clarifying what role, if any, 5-hmC downregulation plays in lymphoproliferative disorders.

From a diagnostic point of view, both the reactivity and atypical presentation of CD4^+^ lymphocytes during the inflammation process can be misdiagnosed as pseudolymphoma. The present data showed that the absence of 5-hmC staining was highly suggestive of PC-SMTLD. In this case, the use of 5-hmC staining could be a useful adjunct to differentiating between PC-SMTLD and pseudolymphoma. This is similar to melanocytopathy, where the expression of 5-hmC has been shown to be a potential marker for differentiating malignant melanoma from benign nevi.^12^ However, with the increase of sample size, genome-wide localization, and the application of 5-hmC tissue detection methods, more genome studies such as single-cell sequencing and spatial transcriptome sequencing are needed to further clarify the role of 5-hmC in rare diseases like lymphoid proliferative disorders.

A number of studies have shown that DNA methylation is closely related to the occurrence of various tumors. The Ten-Eleven Translocation Enzyme (TET) and 5-hmC play important roles in DNA cytosine demethylation. Studies^20^ have shown that DNA methylation of tumor suppressor genes has been found in a variety of tumors such as leukemia, colon cancer, lung cancer, breast cancer, gallbladder cancer, and prostate cancer. TET protein and 5-hmC levels were significantly reduced in the malignant tumor, which suggested 5-hmC might be a significant biological marker of a tumor. The level of 5-hmC is related to the degree of differentiation of some tumors and closely related to the prognosis of tumors.^21^ Although different mechanisms may affect 5-hmC reduction in cancer, the mutation of Ten-Eleven Translocation Enzyme-2 (TET2) is responsible for the reduction of 5-hmC levels in T-Cell Lymphoma and myeloproliferative tumors.^8^, ^9^, ^22^ The reduction of 5-hmC levels in CD4^+^ lymphoproliferative diseases may be an indicator of TET2 mutations. Thus the authors examined both 5-hmC and TET2 expression levels in PC-SMTLD and PL in the immunohistochemical experiments, aiming to explore the changes of 5-hmC and the enzyme TET2 that catalyzes the oxidation of 5-mC. Finally, it was found that the expression level of 5-hmC in PC-SMTLD was lower than PL, while TET2 expression was not different. One possible reason was the change in TET2 activity rather than the expression level.

In some malignancies, the loss of 5-hmC seems to be associated with a more aggressive course and poor prognosis. By contrast, PC-SMTLD is a type of lymphoproliferative disease with undetermined benign or malignant potential, often with a benign course and a good prognosis. The reduction of 5-hmC in PC-SMTLD suggests that the 5-hmC reduction may not be limited to malignant and invasive tumors, or there may be other mechanisms to counteract the effect of 5-hmC reduction so that the disease presents a benign process.

Early descriptions of this entity were classified as peripheral T-cell lymphoma, not otherwise specified (PTCL-NOS). In 2008, the WHO classified PTCL-NOS as “primary cutaneous CD4 positive small/medium T-cell lymphoma”. Although some cases were fatal, the prognosis in many cases was surprisingly good, and some studies have confirmed this preliminary observation. After 8 years of follow-up, the authors found that these lymphomas were indeed indolent or had an undetermined malignant potential, so it is doubtful that they were defined as genuine lymphomas. New changes were made in 2016 WHO new classification of cutaneous lymphomas, with the establishment of new temporary entities designating metastatic entities as lymphomas and the renaming of entities with undetermined Malignant Potential as Lymphoproliferative Diseases(PC-SMTLD). As experience in this area continues to accumulate, it is expected that new temporary entities will emerge from existing ones.

At the Yale Cancer Center, they note the heterogeneity of PC-SMTLD,^23^ suggesting that it is important to distinguish indolent PC-SMTLD cases from more aggressive cases because of the wide differences in prognosis and treatment between these groups. In reported cases, it has been agreed that patients with a single lesion and the histological and immunophenotypic characteristics described above have a good prognosis, with an estimated 5-year survival rate of 75%. Therefore, it should be treated with local modalities in most cases. However, staging assessment is essential to eliminate systemic involvement cases. In rare cases where invasive characteristics are unknown, cytotoxic chemotherapy and involved field radiotherapy can be used as systemic early primary cutaneous T-cell lymphoma. In this experiment, the authors studied the 5-hmC expression of PC-SMTLD and PL, Because of the difference in expression of 5-hmC, 5-hmC can be used as one of the differential diagnostic indicators for PC-SMTLD and PL, which also makes 5-hmC a new potential biological marker for the diagnosis of PC-SMTLD, and also explains the reason why part of PC-SMTLD cases have malignant potential, so as to better diagnose PC-SMTLD with a malignant tendency in the early stage and give early intervention. Thus, stage assessment and treatment can be achieved.

## Conclusion

The authors studied the expression pattern of 5-hmC in pseudolymphoma and lymphoproliferative diseases and found that 5-hmC generally decreased in lymphoproliferative diseases by immunohistochemical staining. Finally, the authors concluded that this epigenetic biomarker was decreased in most cases of lymphoproliferative disease but not in benign cases (pseudolymphoma).

## Financial support

This study was supported by “The World-Class Universities (Disciplines) and The Characteristic Development Guidance Funds for the Central Universities” (Grant nº PY3A0241001016), 10.13039/501100001809National Natural Science Foundation of China (Grant nº 81372912).

## Authors’ contributions

Jiahui Hu: Conceived the ideas; Designed and directed the experiments; data collection, analysis, and interpretation; statistical analysis; critical literature review; writing and approval of the manuscript.

Sonemei Geng: Analysis, and data interpretation; writing; critical literature review and critical review of the manuscript; approval of the final version of the manuscript.

Xingyue Zhang: Approval of the final version of the manuscript; data collection, analysis, and interpretation; manuscript critical review.

Lihong Zhao: Data interpretation; writing; critical literature review and critical review of the manuscript; approval of the final version of the manuscript.

Qiang Zhao: Data interpretation; critical review of the manuscript; approval of the final version of the manuscript.

## Conflicts of interest

None declared.
