# Supplementary material for: Decrease of 5-hydroxymethylcytosine in primary cutaneous CD4+ small/medium sized pleomorphic T-cell lymphoproliferative disorder
Source: An Bras Dermatol. 2023 Aug 30;99(1):27–33. doi: 10.1016/j.abd.2023.01.003 (PMC10964357; doi:10.1016/j.abd.2023.01.003)
Supplement: Supplementary file 1 [file mmc1.docx]

ABD-D-22-00572_Supplementary Material

**Table S1** The clinical data of primary cutaneous CD4^+^ small/medium sized pleomorphic T-cell lymphoproliferative disease.

| **Case** | **Gender** | **Age** | **Location** | **Clinical manifestation** | **Tumor diameter(cm)** | **Therapy** | **Prognosis** | **Follow-up time(month)** |
| --- | --- | --- | --- | --- | --- | --- | --- | --- |
| 1 | Male | 36 | Nasal ala | Solitary red nodule for 21 days | 2 | Excision | Survival without recurrence | 3 |
| 2 | Male | 62 | Nasal ala | Solitary red nodule for 10 days | 1.5 | Excision | Survival without recurrence | 14 |
| 3 | Female | 25 | Cheek | Solitary red nodule for 30 days | 3 | Excision | Survival without recurrence | 14 |
| 4 | Female | 52 | Nasal ala | Solitary red nodule for 28 days | 1 | Excision | Survival without recurrence | 13 |
| 5 | Female | 32 | Nasal tip | Solitary red nodule for 40 days | 2 | Excision | Survival without recurrence | 12 |
| 6 | Male | 78 | Left lower extremity | Lump for 7 years with rupture and exudation for 6 months | 10 | Cyclophosphamide and radiotherapy | Survival without recurrence | 12 |

**Table S2** The clinical and immunohistochemistry data of pseudolymphoma.

| **Case** | **Gender** | **Age** | **Location** | **Clinical manifestation** | **Immunohistochemistry** | **Therapy** | **Prognosis** | **Follow-up time (year)** |
| --- | --- | --- | --- | --- | --- | --- | --- | --- |
| 1 | Male | 23 | Left cervical region | Red papule for 5 years | Positive: CD3, CD45RO, CD20. Ki67 (5%) | Topical glucocorticoid | Survival | 5 |
| 2 | Male | 54 | Cheek | Red nodules for 1 year | Poitive: CD3, CD45RO, CD20, CD79a, Bcl-2, Bcl-6. Ki67 (60%) | Topical glucocorticoid | Survival | 5 |
| 3 | Male | 65 | Cheek | Red nodule for 2 years | Positive: CD3, CD45RO, CD20, LCA. Ki67 (>15%) | Excision | Survival | 5 |
| 4 | Female | 35 | Right arm | Red plaque for 1 year | Positive: CD3, CD45RO, CD5, CD20. | Excision | Survival | 4 |
| 5 | Female | 66 | Trunk | Erythema and papules for 3 years | Positive: CD3, CD5, CD7, CD20, CD79a, LCA. Ki67 (30%) | Topical glucocorticoid | Survival | 4 |
| 6 | Male | 83 | Right arm and trunk | Red nodules for 1 year | Positive: CD3, CD45RO, CD20, CD79a, Bcl-2, Bcl-6, Mum-1. | Systemic and topical glucocorticoid | Survival | 4 |
| 7 | Female | 38 | Cheek | Red plaques for 4 years | Positive: CD45RO, CD20, CD79a, Bcl-2. | Topical glucocorticoid | Survival | 3 |
| 8 | Male | 44 | Nasal ala | Red papule for 2 months | Positive: CD3, CD4, CD45RO, CD8, CD20, CD79a. | Excision | Survival | 3 |
| 9 | Female | 42 | Cheek | Nodule for 2 months | Positive: CD3, CD45RO, CD5, CD20, CD79a, Bcl-2, Bcl-6. Ki67 (10%) | Topical glucocorticoid | Survival | 3 |
| 10 | Female | 77 | Nasal ala | Red plaque for 1 month | Positive: CD3, CD45RO, CD5, CD7, CD20, CD30, CD79a, LCA. | Excision | Survival | 2 |
| 11 | Female | 52 | Nasal tip | Red nodule for 1 year | Positive: CD3, CD45RO, CD7, CD8, CD20, CD30, CD79a. | Excision | Survival | 2 |
| 12 | Male | 17 | Right lower extremity | Red plaque for 7 years | Positive: CD3, CD45RO, CD8, CD20, CD79a. Ki67 (>50%) | Excision | Survival | 2 |
| 13 | Female | 48 | Chest | Red nodule for 1 year | Positive:CD3,CD20. | Excision | Survival | 1 |
| 14 | Male | 30 | Left lower extremity | Red plaque for 1 month | Positive: CD3, CD4, CD8, CD79a, LCA. Ki67 (20%) | Excision | Survival | 1 |
| 15 | Male | 49 | Cheek | Red nodule for 4 years | Positive: CD3, CD45RO, CD20, CD79a, Bcl-2, Bcl-6, Mum-1. Ki67 (15%) | Excision | Survival | 3 |
| 16 | Male | 54 | Nasal tip | Red nodule for 6 months | Positive: CD3, CD4, CD7, CD8, CD20, CD30, CD79a. | Topical glucocorticoid | Survival | 3 |
| 17 | Female | 49 | Cheek | Nodule for 10 months | Positive: CD3, CD20, CD30. | Excision | Survival | 2 |
| 18 | Male | 32 | Lower extremity | Nodule for 2 years | Positive: CD3, CD7, CD8, CD20, CD30, Bcl-2. | Topical glucocorticoid | Survival | 4 |
| 19 | Male | 45 | Nasal tip | Red nodule for 1 year | Positive: CD3, CD45RO, CD7, CD20. | Excision | Survival | 2 |
| 20 | Female | 58 | Trunk | Red nodule for 2 years | Positive: CD3, CD45RO, CD8, CD30. | Excision | Survival | 3 |

**Table S3** The immunohistochemistry data of PC-SMTLD case series.

| **Patient** | **CD3** | **CD4** | **CD7** | **CD8** | **CD20** | **CD30** | **CD56** | **TIA-1** |
| --- | --- | --- | --- | --- | --- | --- | --- | --- |
| 1 | + | + | + | - | +/- | - | - | +/- |
| 2 | + | + | + | - | - | - | - | +/- |
| 3 | + | + | + | +/- | +/- | - | - | N/A |
| 4 | + | + | + | - | - | - | - | - |
| 5 | + | + | N/A | + | - | - | - | + |
| 6 | + | + | N/A | - | - | - | - | N/A |

N/A, Not Available.

**Figure S1 Histological Findings of PL.** Nodular lymphocytic infiltrates was observed in the entire dermis. At high magnification, lymphoid follicle are found. (Hematoxylin & eosin, scale bar = 100 μm).
