# Supplementary figures and images for: Decrease of 5-hydroxymethylcytosine in primary cutaneous CD4+ small/medium sized pleomorphic T-cell lymphoproliferative disorder
Source: An Bras Dermatol. 2023 Aug 30;99(1):27–33. doi: 10.1016/j.abd.2023.01.003 (PMC10964357; doi:10.1016/j.abd.2023.01.003)

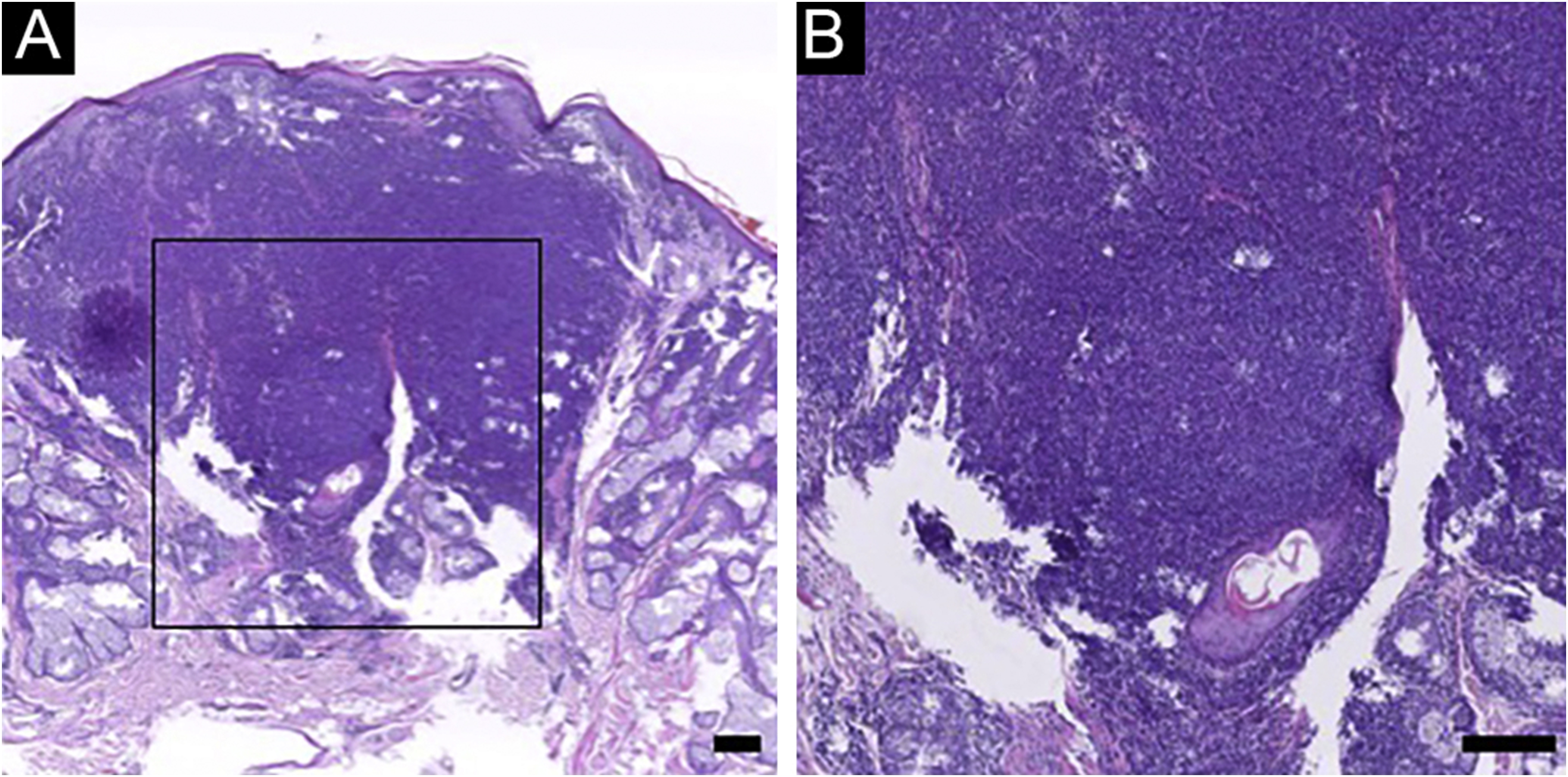

Supplement: Supplementary file 2 [file mmc2.jpg]
